# Ethylene positively regulates cold tolerance in grapevine by modulating the expression of ETHYLENE RESPONSE FACTOR 057

**DOI:** 10.1038/srep24066

**Published:** 2016-04-04

**Authors:** Xiaoming Sun, Tingting Zhao, Shuheng Gan, Xiaodie Ren, Linchuan Fang, Sospeter Karanja Karungo, Yi Wang, Liang Chen, Shaohua Li, Haiping Xin

**Affiliations:** 1 Key Laboratory of Plant Germplasm Enhancement and Specialty Agriculture, Wuhan Botanical Garden, Chinese Academy of Sciences, Wuhan 430074, P.R. China; 2 Sino-Africa Joint Research Center, Chinese Academy of Sciences, Wuhan 430074, P.R. China; 3 Beijing Key Laboratory of Grape Sciences and Enology, CAS Key Laboratory of Plant Resources, Institute of Botany, Chinese Academy of Sciences, Beijing 100093, P.R. China; 4 University of Chinese Academy of Sciences, Beijing 100049, P.R. China

## Abstract

Ethylene (ET) is a gaseous plant hormone that plays essential roles in biotic and abiotic stress responses in plants. However, the role of ET in cold tolerance varies in different species. This study revealed that low temperature promotes the release of ET in grapevine. The treatment of exogenous 1-aminocyclopropane-1-carboxylate increased the cold tolerance of grapevine. By contrast, the application of the ET biosynthesis inhibitor aminoethoxyvinylglycine reduced the cold tolerance of grapevine. This finding suggested that ET positively affected cold stress responses in grapevine. The expression of *VaERF057*, an ET signaling downstream gene, was strongly induced by low temperature. The overexpression of *VaERF057* also enhanced the cold tolerance of *Arabidopsis*. Under cold treatment, malondialdehyde content was lower and superoxide dismutase, peroxidase, and catalase activities were higher in transgenic lines than in wild-type plants. RNA-Seq results showed that 32 stress-related genes, such as *CBF1*-*3*, were upregulated in *VaERF057*-overexpressing transgenic line. Yeast one-hybrid results further demonstrated that *VaERF057* specifically binds to GCC-box and DRE motifs. Thus, *VaERF057* may directly regulate the expression of its target stress-responsive genes by interacting with a GCC-box or a DRE element. Our work confirmed that ET positively regulates cold tolerance in grapevine by modulating the expression of *VaERF057*.

Ethylene (ET) is a gaseous plant hormone that regulates many aspects of the plant life cycle, including seed germination, leaf senescence, fruit ripening, abscission, and biotic and abiotic stress responses^1^,^2^,^3^. ET biosynthesis is mainly regulated by 1-aminocyclopropane-1-carboxylate synthase (ACS) and 1-aminocyclopropane-1-carboxylate oxidase (ACO) at transcriptional or post-translational levels^4^,^5^. In *Arabidopsis*, ET can be captured by a five-member endoplasmic reticulum (ER)-localized receptor family, including ETHYLENE RESPONSE1 and 2 (ETR1 and ETR2), ETHYLENE RESPONSE SENSOR1 and 2 (ERS1 and ERS2), and ETHYLENE INSENSITIVE4 (EIN4). These receptors remove the block of CONSTITUTIVE TRIPLE RESPONSE1 (CTR1) on EIN2^6^,^7^,^8^. EIN2 becomes released and then activates EIN3/EIN3-Like1, which positively regulates the expression of ETHYLENE RESPONSE FACTOR (ERF) transcription factors, such as ERF1^9^,^10^,^11^. These ERF transcription factors participate in developmental and stress-related signal pathways by regulating the expression of downstream genes^12^.

Low temperature is a primary environmental factor that limits plant growth and development, productivity, and geographical distribution^13^. Plants must adjust to various physiological and biochemical processes in response to cold stress^14^. Endogenous ET levels altered under cold stress have been observed in many plant species. However, the role of ET in cold tolerance varies in different plant species, even in *Arabidopsis* grown in different environments. For instance, the cold tolerance of soil-grown *Arabidopsis* seedlings treated with 1-aminocyclopropane-1-carboxylate (ACC) is enhanced^15^ but is reduced *in vitro*; cold tolerance is increased when aminoethoxyvinylglycine (AVG) is applied^14^. ET positively affects the cold tolerance of tomato (*Lycopersicon esculentum*)^16^. By contrast, ET levels are negatively correlated with the cold tolerance of *Medicago truncatula*^17^. ET also negatively influences the cold tolerance of tobacco (*Nicotiana tabacum*), whereas AVG application enhances cold tolerance^18^. Therefore, the role of ET on cold tolerance is possibly species dependent^19^. As such, the function of ET in different species under cold stress should be investigated to elucidate the diverse roles of the ET signal transduction pathway in plants during cold stress.

ERF transcription factors, which are located downstream of the ET signal pathway, belong to the APETALA2/ERF (AP2/ERF) family. This family can be divided into AP2, C-repeat binding factor/dehydration-responsive element binding factor (CBF/DREB), ERF, RAV, and other subfamilies on the basis of their sequence similarities and number of AP2/ERF domains^20^. The ERF subfamily proteins contain single AP2/ERF domain and mainly participate in responses to biotic stresses, such as pathogenesis, by binding to the GCC-box present in the promoter of ET-inducible pathogenesis-related (PR) genes^21^,^22^. *ERF* genes can recognize dehydration-responsive element (DRE) and play an important role in abiotic stress responses^23^. The *ERF* overexpression in *Arabidopsis*, tobacco, and soybean plants positively regulates the expression of several *PR* genes; as a result, the resistances to bacterial, fungal, or viral pathogens of these plants are enhanced^24^,^25^,^26^. *ERF* genes also respond to various abiotic stresses. For instance, overexpression in the sense or antisense orientation of *TERF2/ERF2* can increase or decrease the freezing tolerance of tomato and tobacco, respectively^18^. The overexpression of *GmERF7* enhances the salt tolerance of transgenic tobacco^27^; by contrast, the overexpression of *OsERF3* decreases the drought tolerance of rice^28^. Although many *ERF* genes have been identified in different species, the regulatory pathway of the abiotic stress response of the ERF subfamily remains poorly understood.

Grapevine (*Vitis*) is a widely cultivated fruit crop worldwide. Low temperature is a crucial environmental factor that negatively affects grapevine productivity and quality. To address this problem, researchers widely investigated the genetic mechanisms of cold acclimation in grapevine. Previous studies mainly focused on the CBF pathway in grapevine. The overexpression of *CBF* genes, including *CBF1−4* of grapevines, induces the expression of downstream genes and thus increases the freezing tolerance of transgenic *Arabidopsis* or tobacco plants^29^,^30^,^31^. *CBF* genes belong to the CBF/DREB subfamily of the AP2/ERF family. The AP2/ERF family in grapevine has been subjected to genomic and transcriptomic analyses, and 149 members, including 122 ERF transcription factors, have been identified^32^. Three *ERF* members, namely, *VpERF1, VpERF2*, and *VpERF3* from *V. pseudoreticulata*, are involved in biotic and abiotic stress responses^30^. Xin *et al*.^33^ performed a transcriptomic analysis and identified 16 AP2/ERF transcription factors that respond to low temperature in grapevine. However, the function of ET and its downstream *ERF* genes under low temperature remains unclear.

This study aimed to obtain insights into the function of ET in the cold tolerance of grapevine. A cold-hardy species (*V. amurensis*) and a freezing-sensitive cultivar (*V. vinifera* ‘Muscat Hamburg’) were used to investigate the differences in the ET-related signaling pathway of these two species. ET production under cold treatment was determined in these two grapevine species. The effects of exogenous ET addition and endogenous ET synthesis inhibition on the cold tolerance of grapevine were also evaluated. The function of *VaERF057*, which belongs to the ERF transcription family and is strongly induced by cold stress and exogenous ET, was analyzed in transgenic *Arabidopsis*. RNA-Seq was performed with *VaERF057-*overexpressing *Arabidopsis* transgenic lines to determine the putative downstream genes regulated by *VaERF057*. On the basis of our results, we proposed a comprehensive model of ET-dependent signal transduction in grapevine under cold stress.

## Results

### Low temperature increased ET production in grapevine

**Figure 1:**
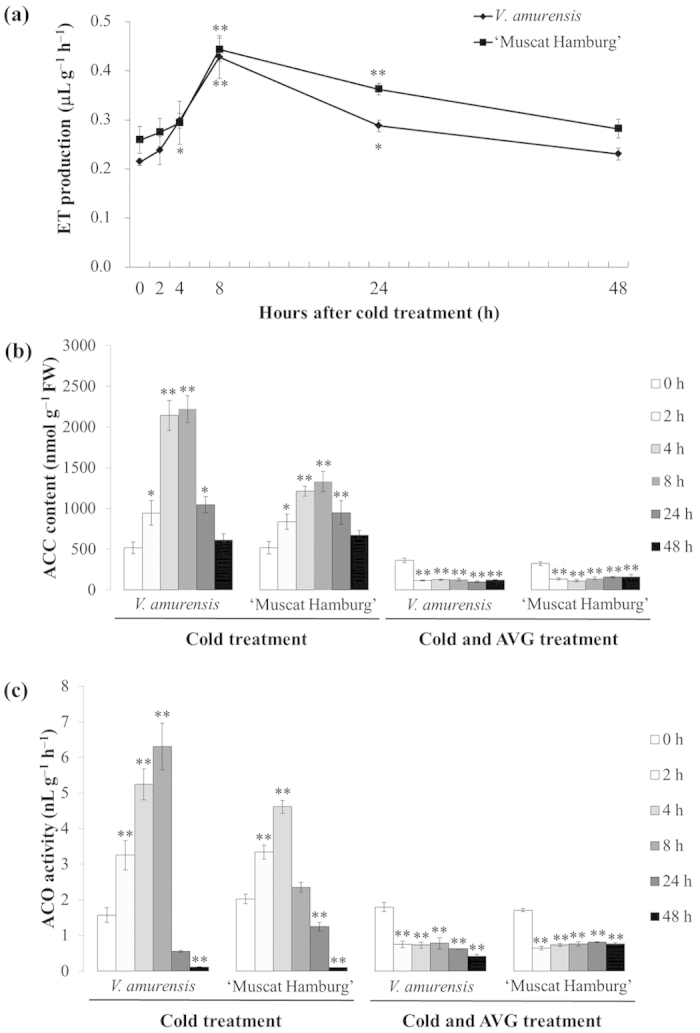
Changes in (**a**) ET production, (**b**) ACC content, and (**c**) ACO activity in *V. amurensis* and ‘Muscat Hamburg’ under cold stress. Determination was performed at 0, 2, 4, 8, 24, and 48 h under cold stress at 4 °C. For AVG treatment, the plantlets were treated with 100 μM AVG under cold stress, and distilled water was used as control. Data are the mean values ± SE of three biological replicates. **and *indicate significant differences compared with the point of 0 h at *P *< 0.01 and *P *< 0.05 (Student’s t-test), respectively.

The function of ET in cold tolerance varies in different plant species. ET biosynthesis increases or decreases under cold stress depending on the plant species^15^,^16^,^34^,^35^. To investigate ET production under cold stress in grapevine, we first determined ET biosynthesis in *V. amurensis* and ‘Muscat Hamburg’ plantlets that were subjected to low temperature (4 °C). A similar evolution of ET production was observed in both species throughout the treatment with low temperature (Fig. 1a). ET production rapidly increased after initiating the cold stress, reached the maximum at 8 h, and then decreased to the original level at 48 h (Fig. 1a). However, the maximum changes in the two grape species were slightly different (1.7-fold in *V. amurensis* and 2-fold in ‘Muscat Hamburg’). These results indicated that ET biosynthesis in grapevine was remarkably enhanced at the early period of cold stress.

ACC is a direct product of ACS and its content can be used as an indicator of ACS activity. ACC content in the two grape species rapidly increased, reached the maximum at 8 h after initiating the cold stress, and then decreased (Fig. 1b). The greatest change in ACC content was up to 4.3-fold in *V. amurensis* and up to 2.6-fold in ‘Muscat Hamburg’. Similar to the changes in ACC content, ACO activity under cold stress first increased and then decreased (Fig. 1c). Moreover, the fold changes in ACO activity were greater in *V. amurensis* than in ‘Muscat Hamburg’. ACO is an enzyme that catalyzes the last step of the ET biosynthetic pathway by converting ACC into ET. The results of the evolution of both ACC content and ACO activity strongly support the increase in ET production in grapevines during the early stage of cold stress.

### Application of ACC and AVG respectively enhanced and reduced the cold tolerance of grapevine

**Figure 2:**
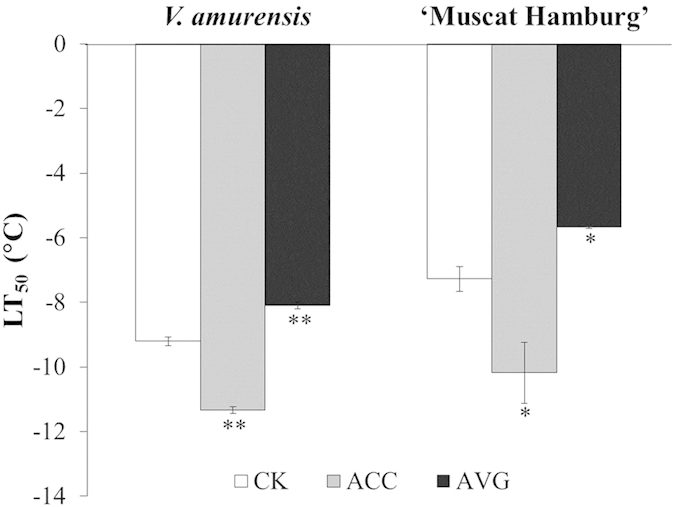
Effects of exogenous application of ACC and AVG on the cold tolerance of *V. amurensis* and ‘Muscat Hamburg’. Plantlets of *V. amurensis* and ‘Muscat Hamburg’ were treated with 100 μM ACC or 100 μM AVG, and distilled water was used as control (CK). LT_50_ values were calculated after the plants were treated with ACC and AVG for 8 h. Data are the mean values ± SE of three biological replicates. **and *indicate significant differences compared with the CK at *P *< 0.01 and *P *< 0.05 (Student’s t-test), respectively.

To establish the relationship between ET production and cold tolerance, we measured the semi-lethal temperature (LT_50_) of grapevine leaves treated with ET precursor (ACC) and ACS inhibitor (AVG). Application of exogenous ACC reduced LT_50_ value from −9.2 °C to −11.3 °C in *V. amurensis* and from −7.3 °C to −10.2 °C in ‘Muscat Hamburg’ (Fig. 2). The application of AVG (100 μM) under cold stress largely inhibited ACC biosynthesis and ACO activity in grapevine (Fig. 1b,c), indicating that AVG can inhibit ET production in grapevine under low temperature. By contrast, application of exogenous AVG increased LT_50_ values to −8.1 °C in *V. amurensis* and −5.7 °C in ‘Muscat Hamburg’ (Fig. 2). Previous results indicated that an increased ET production resulting from exogenous application of ET precursor enhances cold resistance, whereas a reduced cold tolerance was expected from suppression of ET production both in *V. amurensis* and ‘Muscat Hamburg’.

### Isolation and characterization of *VaERF057* and *VvERF057*

**Figure 3:**
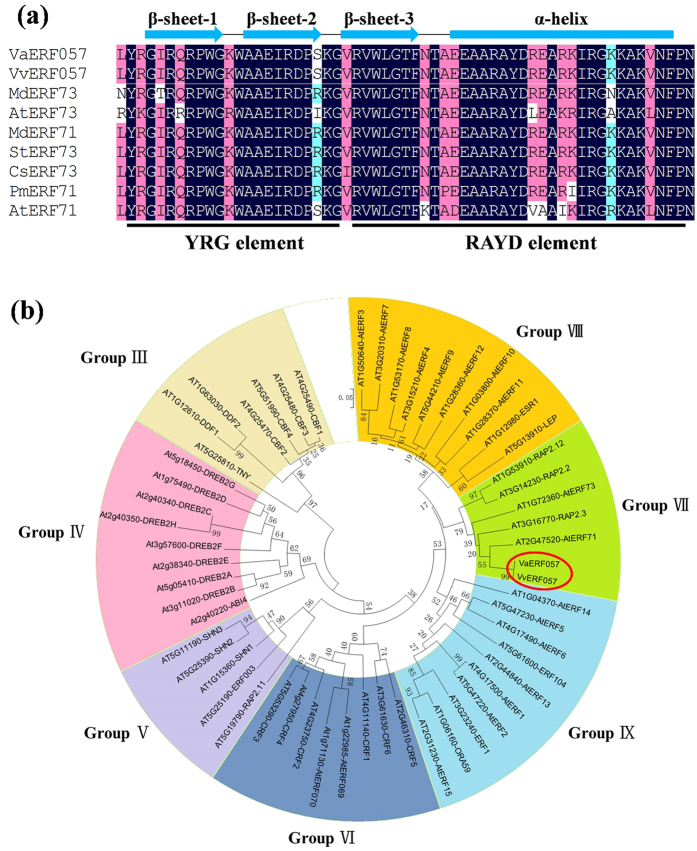
Sequence alignment and phylogenetic analysis of VaERF057 and VvERF057 homologues from different plants. (**a**) Analysis of the conserved AP2/ERF domain of ERF057 homologues from different plants. Three β-sheets and one α-helix of the AP2/ERF domain are marked above their corresponding sequences. The YRG and RAYD elements are indicated below the consensus sequence. The GenBank accession numbers are as follows: *Malus domestica MdERF73* (XP_008369034), *MdERF71* (XP_008386662), *Arabidopsis thaliana AtERF73* (AT1G72360), *ATERF71* (AT2G47520), *Solanum tuberosum StERF73* (XP_006359865), *Cucumis sativus CsERF73* (XP_004152238), and *Prunus mume PmERF71* (XP_008235129). (**b**) Phylogenetic tree of VaERF057 and VvERF057 with ERFs from *Arabidopsis*. The *Arabidopsis* ERF sequences were downloaded from TAIR. The protein sequences were aligned using Clustal W2, and phylogenetic tree was generated by MEGA6.0 software using the neighbor-joining method.

The ERF transcription factors found downstream of the ET signal pathway play an important role in response to abiotic stresses, including salt, drought, and cold stresses^18^,^36^. Our previous study revealed that *ERF057* is significantly upregulated under cold stress^33^. We isolated the *VaERF057* and *VvERF057* genes from *V. amurensis* and ‘Muscat Hamburg’, respectively. The putative protein sequences of VaERF057 and VvERF057 showed 97.5% identity with proteins containing the same AP2/ERF domain (Fig. S1). Analysis of the conserved AP2/ERF domain of 59 amino acids showed that the AP2/ERF domain shares over 90% identity with its homologs from different plant species (Fig. 3a). Based on the conventional classification^37^, the AP2/ERF domain is divided into two conserved segments (YRG and RAYD), and the protein formed contains three β-sheets and a single α-helix (Fig. 3a). Phylogenetic analysis further revealed the evolutionary relationship to the ERFs of *Arabidopsis*, suggesting that VaERF057 and VvERF057 belong to Group VII of the ERF family (Fig. 3b). Given that no significant differences exist between VaERF057 and VvERF057, the functional studies described below mainly focused on VaERF057.

### Increased expression levels of *VaERF057* and *VvERF057* were dependent on enhanced ET production under cold stress

**Figure 4:**
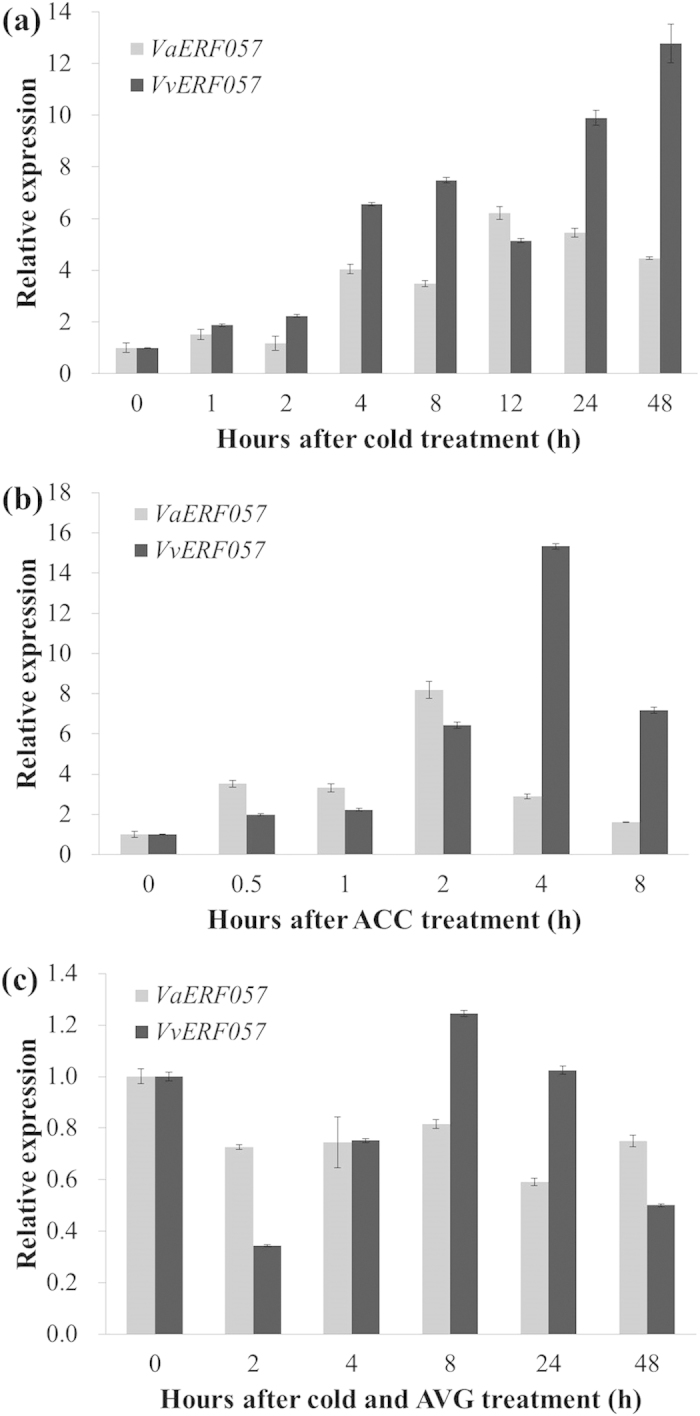
Expression analyses of *VaERF057* and *VvERF057* under (**a**) cold, (**b**) ACC, and (**c**) cold + AVG treatment. *Malate dehydrogenase* gene (*MDH*, GenBank accession number: EC921711) and *β-actin* (GenBank accession number: EC969944) were used as internal controls. Each reaction was replicated three times for each biological sample, using a total of three biological replicates. The expression level of *VaERF057* and *VvERF057* was normalized to that of the internal controls. For each treatment, gene expression at 0 h was set at 1.0 and the expression levels in other time points were calculated accordingly. The relative expression and standard errors for each sample were calculated using Biogazelle qbasePLUS. Error bars represent the standard deviations of nine PCR replicates of three biological replicates.

The expression patterns of *VaERF057* and *VvERF057* in response to cold, ACC and AVG were analyzed by quantitative real time RT-PCR (qRT-PCR). Transcript levels of *VaERF057* and *VvERF057* rapidly increased after initiating cold stress and peaked at 12 h (6.2-fold) and 48 h (12.8-fold), respectively (Fig. 4a). Expression of *VaERF057* and *VvERF057* also rapidly increased in the plants after ACC application (Fig. 4b). The expression of *VaERF057* increased by 8.2-fold at 2 h and *VvERF057* increased by 15.3-fold at 4 h. The effects of cold stress on the expression of *VaERF057* and *VvERF057* were totally inhibited when the plants were subjected with AVG under cold condition (Fig. 4a,c). These results suggested that the response of *VaERF057* and *VvERF057* possibly relies on the increased ET release during cold stress.

### Overexpression of *VaERF057* enhanced cold tolerance of transgenic *Arabidopsis* by reducing malondialdehyde (MDA) content and by increasing superoxide dismutase (SOD), peroxidase (POD), and catalase (CAT) activities

**Figure 5:**
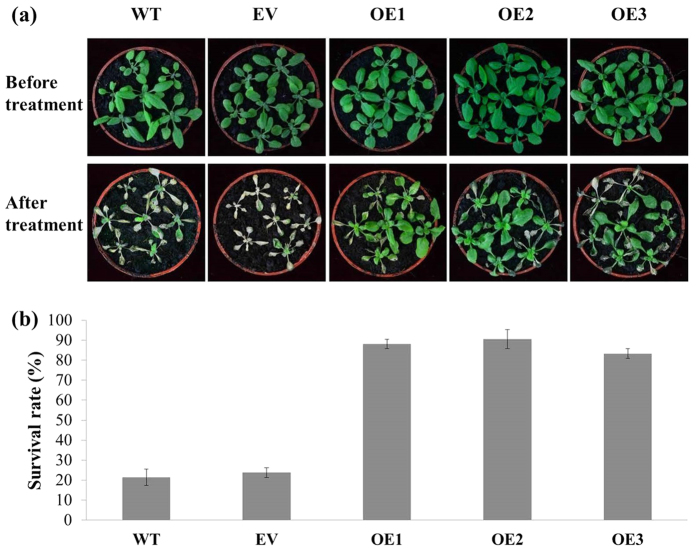
*VaERF057* overexpression conferred enhanced cold tolerance in *Arabidopsis*. (**a**) Phenotypes of the overexpression lines (OE1, OE2, and OE3) and controls [wild type (WT) and empty vector (EV)] after cold treatment. (**b**) Survival rate of *Arabidopsis* after cold treatment.

To further investigate the possible role of *VaERF057* in abiotic stress responses, we generated 10 T3 homozygous transgenic *Arabidopsis* lines, which were confirmed by genomic DNA PCR and qRT-PCR (data not shown). Three representative transgenic lines (OE1, OE2, and OE3) displaying high *VaERF057* expression levels were used in subsequent experiments. *VaERF057-*overexpressing *Arabidopsis* plants displayed significantly improved cold tolerance (Fig. 5a). The average survival rate of the *VaERF057-*overexpressing plants was over 85% after cold stress at −11 °C, whereas that of the wild-type (WT) and empty vector (EV) plants demonstrated only 21.4% and 23.8% survival rate, respectively (Fig. 5b).

**Figure 6:**
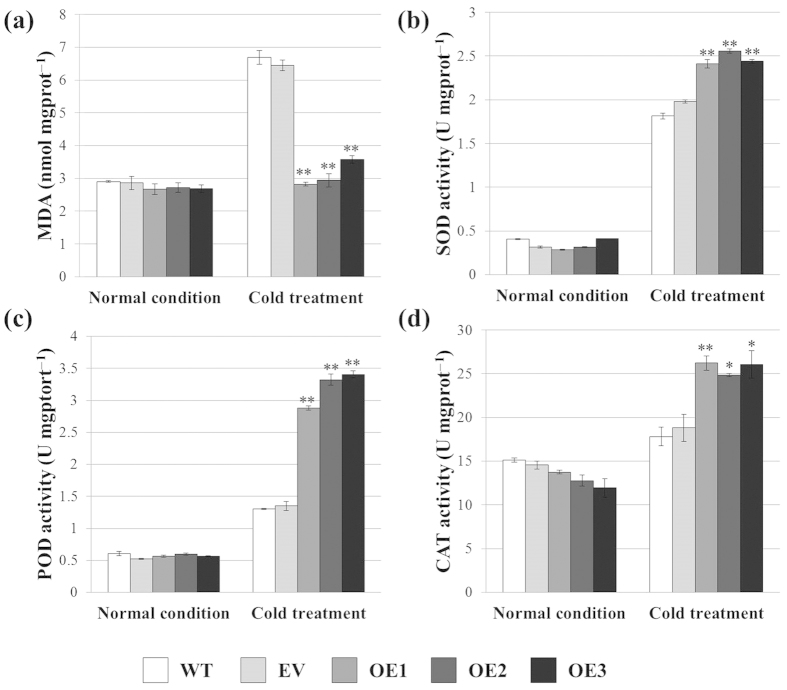
Comparison of the (**a**) MDA content, (**b**) SOD, (**c**) POD, and (d) CAT activities of the overexpression lines (OE1, OE2, and OE3) and controls [wild type (WT) and empty vector (EV)] exposed to cold stress. Data are mean values ± SE of three biological replicates. **and *indicate significant differences compared with the WT at *P* < 0.01 and *P* < 0.05 (Student’s t-test), respectively.

To further investigate the physiological changes under cold treatment in the *VaERF057-*overexpressing *Arabidopsis*, we determined the four important physiological indexes, namely, MDA, SOD, POD, and CAT, which reflect plant cold tolerance. No significant differences were observed among transgenic lines and the controls (WT and EV) under normal conditions (Fig. 6). However, under cold stress, MDA content was significantly lower in transgenic plants than in WT and EV plants (Fig. 6a), whereas SOD, POD, and CAT activities were significantly increased in the transgenic plants compared with that in the WT and EV plants (Fig. 6b–d). These results suggested that the tissues of the *VaERF057-*overexpressing plants were less injured as reflected in their low MDA index, and this phenomenon should increase SOD, POD, and CAT activities to improve tolerance of *Arabidopsis* to cold stress.

### Transcriptome analysis of the *VaERF057-*overexpressing *Arabidopsis* revealed altered expression of genes involved in biotic and abiotic stress responses

To determine the possible direct or indirect targets of *VaERF057*, we performed whole transcriptome sequencing (RNA-Seq) of two biological replicates of WT and three transgenic *Arabidopsis* lines mixture (OE1, OE2, and OE3) under normal conditions. A total of 140 significantly differentially expressed genes demonstrating changes of more than 2-fold were found in the transgenic lines compared with the WT plants. Among these altered genes, 89 were upregulated and 51 were downregulated (Table S2). To confirm the RNA-Seq results, we verified the 23 significantly differentially expressed genes, including 16 upregulated and 7 downregulated genes by using qRT-PCR (Fig. S3). All of these verified genes showed similar changes in expression as revealed by RNA-Seq and qRT-PCR, indicating the reliability of the transcription profile analysis.

To gain insight into the functions of these genes, we performed Gene Ontology (GO) term enrichment analysis by using agriGO v1.2 in default parameters^38^. The most significantly enriched biological processes among these significantly differentially expressed genes in transgenic *Arabidopsis* are involved in responding to stimuli (including biotic and abiotic stimuli, cold, heat, water deprivation, hypoxia, wounding, pathogen, and osmotic and oxidative stresses), metabolic processes, biological regulation, and signal transduction (Fig. S2).

Among the significantly differentially expressed genes, 39 genes are involved in cold, drought, salt, hypoxia, and pathogen stresses; 32 of these genes were upregulated and 7 were downregulated (Table 1). Many key genes involved in these stress tolerances were identified. For instance, *CBF1, CBF2*, and *CBF3*, which encode a small family of transcriptional activators that play an important role in freezing tolerance and cold acclimation in *Arabidopsis*^39^,^40^, were upregulated by 3.9-, 2.4-, and 4.5-fold, respectively. *NCED3* (upregulated by 2.7-fold) is a key gene in the ABA biosynthesis pathway and enhances plant tolerance to abiotic stresses by regulating endogenous ABA biosynthesis^41^,^42^. These results showed that *VaERF057* overexpression can regulate expression of many genes involved in biotic and abiotic stress response. The enhanced cold tolerance of the *VaERF057-*overexpressing *Arabidopsis* suggested that *VaERF057* directly or indirectly regulates expression of these stress-responsive genes.

### VaERF057 demonstrated binding activity to GCC-box and DRE motifs

**Figure 7:**
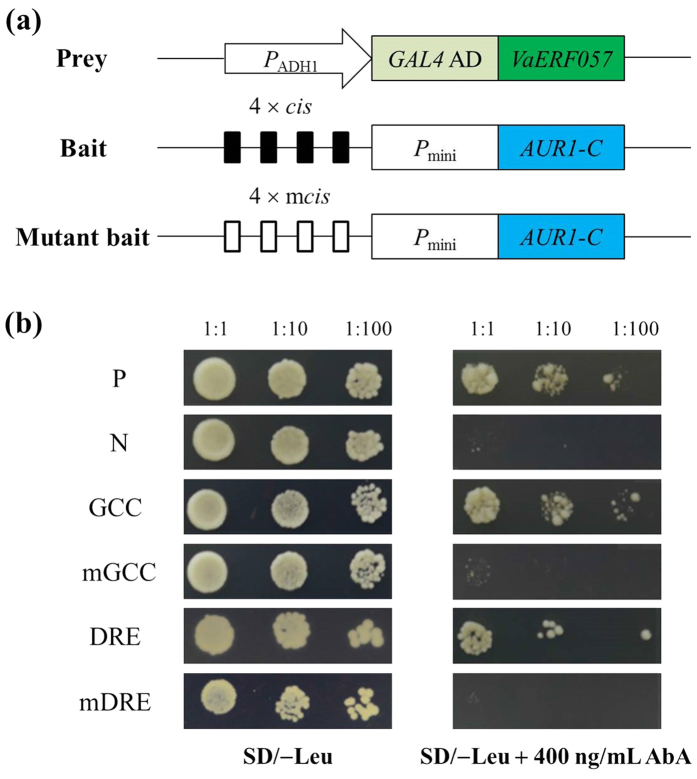
Analyses of the binding activity of VaERF057 to GCC-box and DRE motifs. (**a**) Diagram of prey and bait vectors. VaERF057 protein was fused with the GAL4 AD in a pGADT7-AD vector to generate the prey (pGAD-VaERF057). Four tandem copies of the GCC-box, DRE, or their corresponding mutants were cloned into the pAbAi vector and used as baits. (**b**) Positively co-transformed yeast cells were determined by spotting serial dilutions (1:1, 1:10, and 1:100) of yeast on SD/−Leu medium supplemented with 400 ng ml^−1^ AbA. P: positive control (pGAD-p53 + p53-AbAi); N: negative control (pGADT7-AD + p53-AbAi).

Previous studies have shown that some ERF proteins can bind to GCC-box or DRE motif^24^,^25^,^43^. Especially, *AtERF71/HRE2*, the closest homologue of *VaERF057* in *Arabidopsis*, can bind to both GCC-box and DRE motifs^43^. To determine whether VaERF057 can also bind to GCC-box and DRE motifs, we cloned four tandem copies of GCC-box, DRE, or their mutants into pAbAi vector, which was interacted with the pGADT7 activation domain vector harboring VaERF057 by using Y1H system. Figure 7 shows that VaERF057 can specifically bind to both GCC-box and DRE motifs but failed to bind to all of the mutated GCC-box and DRE motifs. Among the 140 significantly differentially expressed genes, 24 genes contain GCC-box and 54 genes contain at least one DRE core motif upstream of their 1 kb promoter region (Table S2). These results suggested that VaERF057 regulates the expression of target genes by binding to GCC-box and DRE motifs in their promoter regions.

## Discussion

This study explored the function of ET and the roles of its downstream gene *VaERF057* in grapevine during cold stress. Our results demonstrated that cold stress enhanced the production of ET and the expression of ET signal pathway downstream genes, such as *VaERF057* and *VvERF057*, in grapevine. Enhanced ET synthesis is essential in cold tolerance both in cold-hardy (*V. amurensis*) or cold-sensitive (*V. vinifera* ‘Muscat Hamburg’) grapevine.

Our findings are consistent with previous results^15^,^16^,^18^,^35^ wherein an increase of ET level positively regulates the cold tolerance of grapevine. In addition, ET biosynthesis is required for the accumulation of antifreeze proteins in winter rye (*Secale cereale*) during cold acclimation^44^. ET has also been proposed to protect mitochondrial activity in *Arabidopsis* under cold stress^45^. However, an opposite conclusion was reached in other studies on several plant species, including *Arabidopsis*^14^, *M. truncatula*^17^, winter wheat (*Triticum aestivum*)^34^, and dwarf bean (*Phaseolus vulgaris*)^46^. Especially in Arabidopsis, Catala *et al*.^15^ reported that ET production increases under cold treatment and that ET positively regulates cold tolerance, whereas Shi *et al*.^14^ obtained the opposite results. These different results can be largely caused by the different growth conditions employed in these studies. Catala *et al*.^15^ used soil-grown plants, whereas Shi *et al*.^14^ used seedlings grown on Murashige and Skoog (MS) medium in Petri dishes, implying a growth condition of high relative humidity. Indeed, ET production is inhibited in response to cold stress under high relative humidity^47^,^48^. In our research, the grapevine plantlets were grown on half-strength MS medium in a conical flask covered with a sealing film that allows good aeration. The relative humidity in our study was higher than the relative humidity the soil-grown plants were exposed to but lower than that to which the plants grown on MS medium in Petri dishes were exposed. Therefore, a critical value of relative humidity possibly inhibits ET production under cold stress. The function of ET in cold stress in different species exposed to different conditions still requires further investigation.

**Figure 8:**
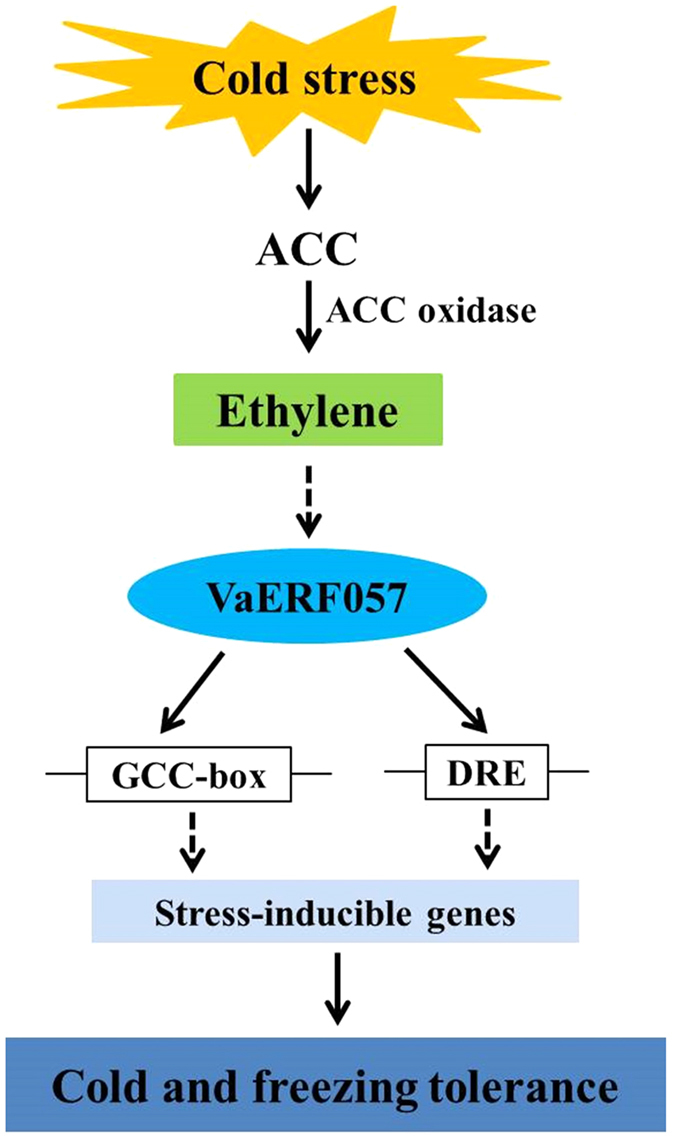
Model of ET-dependent signal transduction through *VaERF057* under cold stress in grapevine. The model indicates that the cold stress-induced increase in ET level activates the ET signaling pathway and upregulates *VaERF057* expression, resulting in transcriptional activation of stress-inducible genes and in enhanced cold tolerance of grapevine.

ERF transcription factors, which are located downstream of the ET signal pathway, were reported to respond to various biotic and abiotic stresses in several species. In *Arabidopsis, AtERF1−5* genes were differentially regulated by ET and by abiotic stresses, such as cold, drought, wounding, high salinity, or pathogen. *AtERF1, AtERF2*, and *AtERF5* function as activators, whereas *AtERF3* and *AtERF4* act as repressors by downregulating not only the basal transcription levels of a reporter gene but also the transactivation activity of other transcription factors^49^,^50^. Many other *ERFs* were identified as stress-responsive through detection of their expression under various stress conditions, although functional studies on *ERF* genes remain limited. Transcript analysis in ‘Cabernet Sauvignon’ grapevines revealed 13 ET–responsive transcripts upregulated and 7 downregulated after cold treatment, whereas *ERF057* wasn’t included^51^. This study isolated the Group VII ERF genes (*VaERF057* and *VvERF057*) from grapevine, and these genes can be induced by cold stress and exogenous ET. AVG can also totally inhibit the inductive effect of cold stress on the expression of these genes. Low temperature promoted the release of ET, and the ET increased the expression of *VaERF057 and VvERF057*. Therefore, the enhanced expression of *VaERF057* under cold stress was possibly directly induced by ET, the release of which was promoted by low temperature (Fig. 8).

As transcription factors, the AP2/ERFs play important roles in stress response by regulating the expression of downstream stress-related genes. For instance, overexpression of *CBF* genes in plants enhances cold tolerance by regulating the expression of cold-responsive genes^52^,^53^,^54^. *TERF2/ERF2* overexpression can also increase cold tolerance in rice, tomato, and tobacco by activating expression of cold-related genes^18^,^55^. In the present study, *VaERF057* overexpression enhanced cold tolerance in transgenic *Arabidopsis*. These physiological adjustments confer cold tolerance to the transgenic *Arabidopsis*. Moreover, RNA-Seq analysis showed that *VaERF057* overexpression in *Arabidopsis* regulated the expression of the cold-induced regulatory genes, including *AtCBF1−3, WRKY33, WRKY70, NCED3*, and *ERF5* under normal condition. An increasing number of evidence indicates that ERF transcription factors play critical regulatory roles in response to stress by interacting with GCC-box or DRE motif to activate expression of targeted stress-responsive genes^24^,^56^,^57^. *AtERF71/HRE2* is the closest homologue of *VaERF057* in *Arabidopsis*. Binding of *AtERF71/HRE2* to GCC-box and DRE motifs was detected by electrophoretic mobility shift assay (EMSA), fluorescence measurement and surface plasmon resonance spectroscopy (BIAcore) experiments^43^. In this study, we provided evidence that *VaERF057* can bind to both GCC-box and DRE motifs by Y1H system. Our results indicate that *VaERF057* in *Arabidopsis* possibly directly upregulates expression of its targeted genes by interacting with these *cis*-elements. The accumulated expression of *VaERF057* and *VvERF057* was also found in other stress conditions, such as drought and high salinity (data not shown). Further studies should be conducted to overexpress or to knock down *VaERF057* and *VvERF057* in grapevine and thus elucidate their functions in stress response.

In summary, low temperature can promote the release of ET and endogenous ET can enhance the cold tolerance of grapevine. We isolated a group VII stress-responsive *ERF* gene (*VaERF057*), which is located downstream of the ET signal pathway and is implicated as a positive regulator of cold tolerance. *VaERF057* overexpression enhanced the cold tolerance of *Arabidopsis*. This finding indicated that *VaERF057*, when induced by ET, may function as a key regulator that improves the cold tolerance of grapevine. *VaERF057* may directly upregulate the expression of its target stress-responsive genes by interacting with GCC-box or DRE/CRT element in grapevine. On the basis of our results, we proposed a comprehensive model of ET-dependent signal transduction in grapevine under cold stress (Fig. 8). Further studies should be performed to determine other components related to *VaERF057* and to gain a clear-cut silhouette of the major hub in the network.

## Methods

### Plant materials and treatments

Micropropagated *V. amurensis* and *V. vinifera* ‘Muscat Hamburg’ plantlets were grown on half-strength Murashige and Skoog (1/2 MS, pH 5.8) solid medium containing 30 g L^−1^ of sucrose and 0.7% agar in conical flasks (120 mL) in a growth chamber at 26 °C (Fig. S3). The average photosynthetic photon flux was 100 μmol m^−2^ s^−1^ with a 16 h light and 8 h dark cycle. Six-week-old plantlets were subjected to cold stress or supplied with plant growth regulators. For cold treatment, whole plants in conical flasks were placed in an illuminated incubation chamber at 4 °C. For treatments with plant growth regulators, plantlet leaves were sprayed with 100 μ μM 1-aminocyclopropane-1-carboxylate (ACC, an ethylene (ET) precursor) or 100 μM aminoethoxyvinylglycine (AVG, an ACC synthase inhibitor), whereas distilled water was used as control. The shoot apex with one well developed leaf was harvested at appropriate times. To reduce the differences between individuals, we collected the samples from three independent replicate plantlets at a single time point, with three biological replicates per treatment. After the collection, all samples were immediately frozen in liquid nitrogen and then stored at −70 °C.

Seeds of *Arabidopsis thaliana* (ecotype Columbia-0, Col-0) were surface-sterilized with 6% sodium hypochlorite for 5 min followed by three washes with sterile water and then plated on 1/2 MS solid medium containing 0.7% agar. One-week-old seedlings were transferred from plates into pots filled with peat moss in a greenhouse under controlled environmental conditions, at 22 °C, under 50–60% relative humidity, and under 130 μmol photons m^−2^ s^−1^ with a 16 h/8 h light/dark cycle.

### Determination of ET production

Six-week-old plants grown in conical flasks (120 ml) under standard conditions (26 °C) or exposed to low temperature (4 °C) were maintained open for 1 h. The conical flasks were subsequently sealed with rubber stoppers to collect ET produced within 1 hour at 0, 2, 4, 8, 24, and 48 h. Gas (1 ml) was withdrawn from each conical flask using a gas-tight syringe and left for 5 min to equilibrate. The gas samples were subsequently injected into a gas chromatograph (Agilent 7890A) equipped with an HP-5 column and a flame ionization detector. The detector and injector were operated at 180 °C and 120 °C, respectively, whereas the oven temperature was set at 110 °C. The carrier gas was nitrogen at a flow rate of 40 ml min^−1^. ET production (μl g^−1^ FW h^−1^) was determined by comparison with an ET standard. All measurements were obtained in triplicate from three independent samples.

### Determination of ACC content

Determination of ACC content was performed according to the method described by Zhao *et al*.^17^ with some minor modifications. In brief, the tissues were ground with a mortar and pestle in 2 ml of 80% ethanol until homogenized. After centrifugation at 12,857 × *g* for 10 min, the supernatant liquid was dried using a centrifugal dryer. The residue was dissolved in 1 ml of deionized water, and 0.3 ml of sample to be assayed was added in a 7 ml vial in an ice bath. Subsequently, 100 μl of HgCl_2_ (10 mM) was added and then the vial was sealed with a rubber serum cap. Afterwards, 100 μl of cold mixtures of 5% NaOCl and saturated NaOH (2:1, v/v) was injected into the vial and the mixture was kept on ice for 30 min. After shaking vigorously, ET production was determined as described above. The efficiency of conversion of ACC into ET was calculated by adding authentic ACC (1 mM) as an internal standard. ACC content was calculated from the quantity of ET liberated and from the conversion efficiency.

### Determination of ACO activity

ACO activity was detected according to the method described by Zhao *et al*.^17^ with some minor modifications. Briefly, the tissues were ground with a mortar and pestle in extraction buffer containing 100 mM Tris (pH 7.2), 30 mM sodium ascorbate, 10% (w/v) glycerol, and 5% (w/v) polyvinylpolypyrrolidone (PVPP). After centrifugation at 18,514 *g* for 30 min at 4 °C, the supernatant liquid was used for enzyme activity analysis. Crude extract (200 μl) was then mixed with 1.7 ml of extraction buffer (without PVPP), 100 μl of FeSO_4_ (1 M), and 100 μl of ACC (20 mM) in a 7-ml capped vial. After incubation at 30 °C for 1 h, ET production was determined as described above.

### Electrolyte leakage assay

Electrolyte leakage was measured to verify the effect of exogenous ACC and AVG treatment on cold tolerance of grapevine. After treatment with ACC (100 μM) and AVG (100 μM) for 8 h, leaf discs were obtained from *V. amurensis* and ‘Muscat Hamburg’ plantlets. The 10 ml tubes containing five leaf discs were placed in a low-temperature illuminated incubation chamber at 0 °C for an hour. The temperature was subsequently reduced at 2 °C h^−1^. The tubes were then removed at −6 °C, −8 °C, −10 °C, and −12 °C for *V. amurensis* and at −4 °C, −6 °C, −8 °C, and −10 °C for ‘Muscat Hamburg’. The leaf discs were thawed overnight at 4 °C in the dark, and 3 ml of deionized water was added into each tube. After shaking at 200 rpm for 2 h at 25 °C, the electrical conductivity of the samples was first measured. The samples were then autoclaved at 121 °C for 20 min, and the final conductivity was measured. Relative electric conductivity is the ratio of initial conductivity before autoclaving to the final conductivity after autoclaving. The semi-lethal temperature (LT_50_) was calculated by logistic equation. Each point was repeated for five times.

### Cloning and sequence analyses of *VaERF057* and *VvERF057*

To clone the *ERF057* gene (*GSVIVT01028050001*) of *V. amurensis* and ‘Muscat Hamburg’, we designed a pair of primers (ERF057-ORF-F/ERF057-ORF-R) based on the sequence of *V. vinifera* ‘Pinot Noir’ (PN40024) genomes (Table S1). The open reading frame (ORF) sequences of *VaERF057* and *VvERF057* were amplified by polymerase chain reaction (PCR). An analysis of the VaERF057 and VvERF057 protein structures was performed using Smart (http://smart.embl-heidelberg.de/). Sequence alignments of the AP2/ERF domain with the other ERFs from different plant species was done by DNAMAN 7.0. Phylogenetic analysis was performed using MEGA6.0 software by using the neighbor-joining method, and the internal branch support was estimated using 1000 bootstrap replicates.

### Gene expression analysis by quantitative real time RT-PCR (qRT-PCR)

Total RNA was extracted from the collected samples by using Column Plant RNAOUT 2.0 Kit (Tiandz, Beijing, China) according to the manufacturer’s instructions. RNase-free DNase I (Promega, USA) was used to degrade any DNA present in the extracted RNA. cDNA was synthesized using SuperScript III Reverse Transcriptase (Invitrogen, USA) with Oligo-dT according to the manufacturer’s instructions. qRT-PCR was performed in an ABI SteopOneplus^TM^ Real-Time PCR System (Applied Biosystems, USA) using FastStart Universal SYBR Green Master (Roche, Shanghai, China). Each reaction was replicated three times for each biological sample, using a total of three biological replicates. The relative expression and standard errors were calculated using Biogazelle qbasePLUS as described in our previous study^58^.

### Generation of overexpression transgenic *Arabidopsis*

The full-length ORF of *VaERF057* containing the restriction sites of Kpn I and Xba I was ligated into pCAMBIA 1301s driven by the CaMV 35S promoter and transformed into *Arabidopsis* using the floral dip method. The positively transformed plants were selected by screening successive generations on hygromycin B (50 mg L^−1^) and confirmed by genomic DNA PCR and qRT-PCR. Three T3 homozygous transgenic lines were selected for further analyses.

### Stress treatments of transgenic *Arabidopsis*

Three-week-old WT and transgenic *Arabidopsis* were subjected under cold tolerance assays. To evaluate cold tolerance, we placed the plants in a growth chamber set at −1 °C for 8 h in the dark. The chamber was then cooled at a rate of 1 °C h^−1^ until −11 °C was reached. After exposure to −11 °C for 3 h, the plants were thawed at 4 °C for 12 h in the dark and then transferred into standard condition. The survival rate was scored 3 days later. Each sample contained 14 seedlings, and each experiment was performed in three biological replicates.

### Physiological analysis of the cold stress response of the *Arabidopsis* plants

For physiological analysis, 3-week-old seedlings of WT and transgenic *Arabidopsis* plants were treated at 4 °C (cold stress) for 4 days, and the plants grown under normal condition served as control. Superoxide dismutase (SOD), peroxidase (POD), and catalase (CAT) activities, along with malondialdehyde (MDA) contents, were measured according to the protocol described by Shin *et al*.^59^. Each sample was pooled from three seedlings, and each experiment was performed in three biological replicates.

### RNA-Seq analysis

RNA-Seq analysis was performed using 3-week-old WT and transgenic *Arabidopsis* mixture under normal condition. Total RNA was extracted using Column Plant RNAOUT 2.0 Kit (Tiandz, Beijing, China) as described above, and mRNA sequencing libraries were constructed using the TruSeq RNA Sample Preparation Kit (Illumina, USA). Sequencing was performed on an Illumina HiSeq3000 platform. Materials of the libraries were pooled from six WT plants and three transgenic *Arabidopsis* lines (OE1, OE2, and OE3). The RNA-Seq experiments were performed in two biological replicates. The total reads were mapped to the TAIR10 *Arabidopsis* genome reference by using the TopHat software^60^. The number of sequencing reads generated from each sample was converted into fragments per kilobase of transcript per million fragments mapped. Significantly differentially expressed transcripts between WT and transgenic lines were detected from two biological replicates by using Cuffdiff with default Benjamini–Hochberg correction for multiple-testing, based on a False Discovery Rate (FDR) <0.05. Functional annotations of genes and AGI symbols were retrieved from TAIR10 datasets. RNA-Seq data were submitted to NCBI and can be accessed under the GEO accession number GSE75456.

### Yeast one-hybrid assay

Yeast one-hybrid assay was performed using the Matchmaker Gold Yeast One-Hybrid Library Screening System (Clontech) to examine the ability of *VaERF057* to bind with GCC-box and DRE motifs. The ORF of *VaERF057* was fused in-frame with the GAL4 activation domain (AD) in a pGADT7-AD vector to generate the prey plasmid (pGAD-VaERF057). A synthesized DNA fragment harboring four tandem copies of the GCC-box, DRE or their mutants, and mGCC or mDRE was cloned into the pAbAi vector to construct the baits. The *BstBI*-cut bait vectors were transformed into the Y1HGold yeast strain. After selecting the transformants on SD/−Ura plates and determining the minimal inhibitory concentration of Aureobasidin A (AbA) for the bait strains, we introduced pGAD-VaERF057 into the Y1HGold strain. Positively co-transformed yeast cells were determined by spotting serial dilutions (1:1, 1:10, and 1:100) of yeast on SD/−Leu medium supplemented with 400 ng ml^−1^ AbA and cultured at 30 °C for 3 days. Positive (pGAD-p53 + p53-AbAi) and negative (pGADT7-AD + p53-AbAi) controls were processed in the same manner.

### Statistical analysis

The reported experimental data represent at least three independent biological repeats. Where appropriate, results are reported as mean values ± standard errors. Mean comparisons were calculated by Student’s t-test, and *P*-values and data sizes were indicated in the figure legends.

## Additional Information

**How to cite this article**: Sun, X. *et al*. Ethylene positively regulates cold tolerance in grapevine by modulating the expression of ETHYLENE RESPONSE FACTOR 057. *Sci. Rep.* **6**, 24066; doi: 10.1038/srep24066 (2016).

## Supplementary Material

Supplementary Information

## Figures and Tables

**Table 1:** Significant differentially expressed genes involved in cold, drought, salt, hypoxia, and pathogen stresses in transgenic *Arabidopsis*.

AGI locus	Symbol	Fold change	FDR	Response to abiotic or biotic stress*				
				Cold	Drought	Salt	Hypoxia	Pathogen
Upregulated genes								
AT5G39890	PCO2	8.05	0.0056				YES	
AT5G54470	BBX29	5.62	0.0056	YES				
AT2G44840	ATERF13	4.81	0.0360		YES	YES		
AT5G47230	ERF5	4.52	0.0056	YES				
AT4G25480	CBF3	4.47	0.0056	YES	YES	YES		
AT2G16060	GLB1	4.36	0.0056				YES	
AT4G25490	CBF1	3.87	0.0273	YES	YES			
AT5G27420	CNI1	3.57	0.0056		YES	YES		
AT2G20880	ERF053	3.46	0.0056		YES	YES		
AT2G38470	WRKY33	3.42	0.0056	YES	YES	YES		YES
AT1G76650	CML38	3.35	0.0056					YES
AT2G40140	CZF1	3.21	0.0056	YES				YES
AT5G54490	PBP1	3.18	0.0056		YES	YES		
AT1G02450	NIMIN1	3.13	0.0498				YES	YES
AT3G02550	LBD41	3.13	0.0056				YES	
AT1G27730	STZ	3.12	0.0056	YES	YES	YES		
AT3G14440	NCED3	2.69	0.0056	YES	YES	YES		
AT1G66090	–	2.62	0.0056					YES
AT5G54610	ANK	2.56	0.0056					YES
AT1G09350	AtGolS3	2.51	0.0391	YES				
AT3G56710	SIB1	2.49	0.0056					YES
AT5G57220	CYP81F2	2.46	0.0102				YES	YES
AT5G15120	PCO1	2.45	0.0441				YES	
AT3G55980	SZF1	2.42	0.0056					YES
AT5G60900	RLK1	2.37	0.0056					YES
AT4G25470	CBF2	2.36	0.0418	YES				
AT3G61890	ATHB-12	2.35	0.0214		YES	YES		YES
AT5G61600	ERF104	2.27	0.0056					YES
AT2G33380	RD20	2.16	0.0056	YES	YES	YES		YES
AT5G20830	SUS1	2.08	0.0056	YES	YES		YES	
AT3G56400	WRKY70	2.06	0.0056	YES			YES	YES
AT1G32920	–	2.01	0.0056					YES
Downregulated genes								
AT1G35140	PHI-1	−7.12	0.0056				YES	
AT2G26020	PDF1.2b	−6.61	0.0214					YES
AT1G21910	DREB26	−3.74	0.0056	YES				
AT2G14610	PR1	−3.49	0.0056		YES			YES
AT4G16260	–	−2.84	0.0056			YES		YES
AT5G57560	TCH4	−2.66	0.0056	YES				
AT3G05727	–	−2.03	0.0056					YES

^*^The annotation of all genes was retrieved from TAIR 10.
