# Supplementary material for: Ethylene positively regulates cold tolerance in grapevine by modulating the expression of ETHYLENE RESPONSE FACTOR 057
Source: Sci Rep. 2016 Apr 4;6:24066. doi: 10.1038/srep24066 (PMC4819186; doi:10.1038/srep24066)
Supplement: Supplementary Information [file srep24066-s1.pdf]

Supplementary information

Ethylene positively regulates cold tolerance in grapevine by modulating the expression of ETHYLENE RESPONSE FACTOR 057

Xiaoming Sun[1, 3, 4], Tingting Zhao[1, 4], Shuheng Gan[1], Xiaodie Ren[1], Linchuan Fang[1], Sospeter Karanja Karungo[1, 4], Yi Wang[3], Liang Chen[1], Shaohua Li[3*] and Haiping Xin[1, 2*]

1. Key Laboratory of Plant Germplasm Enhancement and Specialty Agriculture, Wuhan Botanical Garden, Chinese Academy of Sciences, Wuhan 430074, P.R. China

2. Sino-Africa Joint Research Center, Chinese Academy of Sciences, Wuhan 430074 P.R. China

3. Beijing Key Laboratory of Grape Sciences and Enology, CAS Key Laboratory of Plant Resources, Institute of Botany, Chinese Academy of Sciences, Beijing 100093, P.R. China

4. University of Chinese Academy of Sciences, Beijing 100049, P.R. China

[*]Correspondence author:

SL: shhli@ibcas.ac.cn

HX: xinhaiping215@hotmail.com

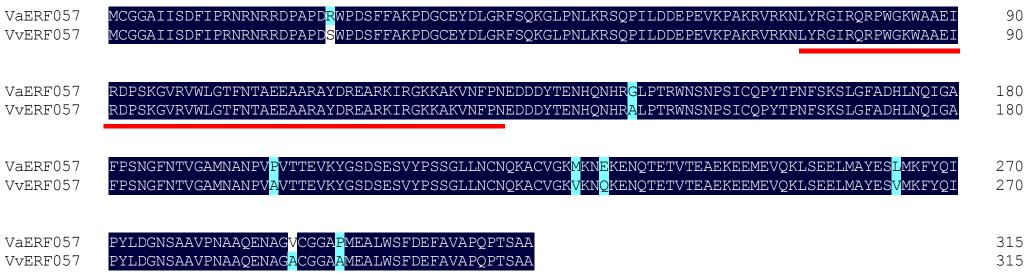

Figure S1. Protein sequence alignment between VaERF057 and VvERF057. The conserved AP2/ERF domains are marked below the corresponding sequences.

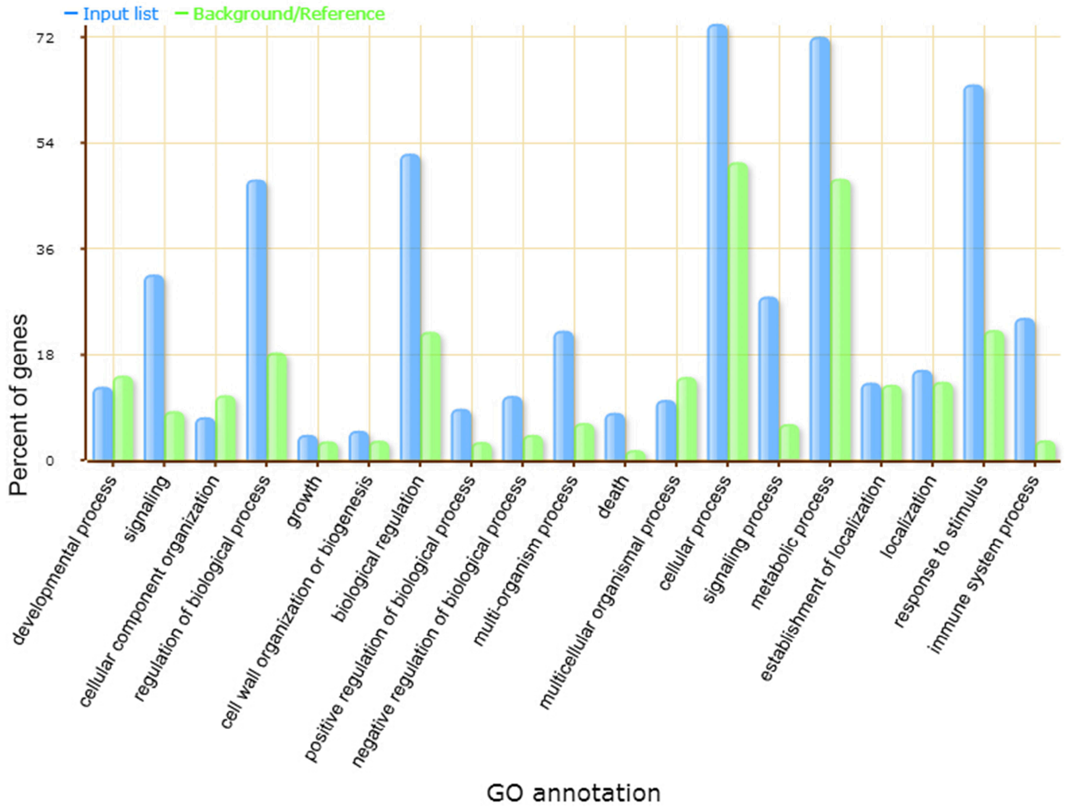

Figure S2. GO term enrichment analysis of significantly differentially expressed genes identified in the *VaERF057* overexpressing Arabidopsis. Blue and green bars indicate the enrichment of target genes in GO terms and the percentage of total annotated Arabidopsis genes mapping to GO terms, respectively.

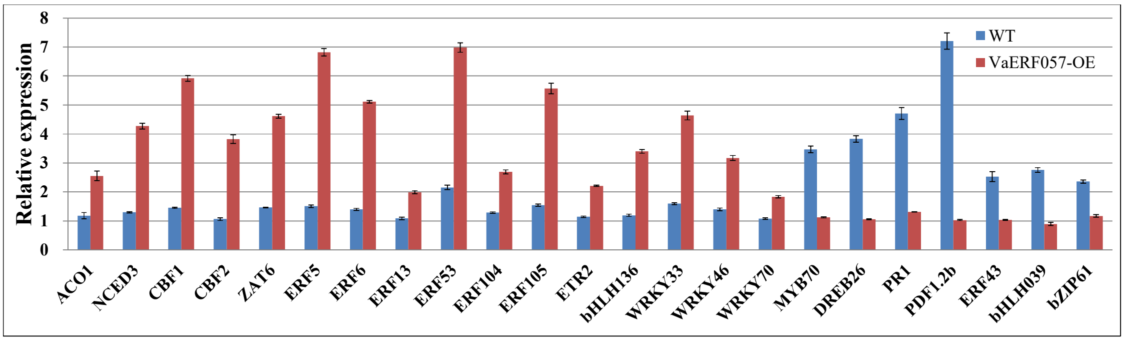

Figure S3. Expression patterns of 23 putative *VaERF057* downstream genes by qRT-PCR analysis. The *ACT2* (GenBank accession number: AT3G18780) and *UBQ10* (GenBank accession number: AT4G05320) were used as the internal controls. Each reaction was replicated three times for each biological sample, using a total of three biological replicates. The relative expression was normalized to that of the internal controls. Error bars represent the standard deviations of nine PCR replicates of three biological replicates.

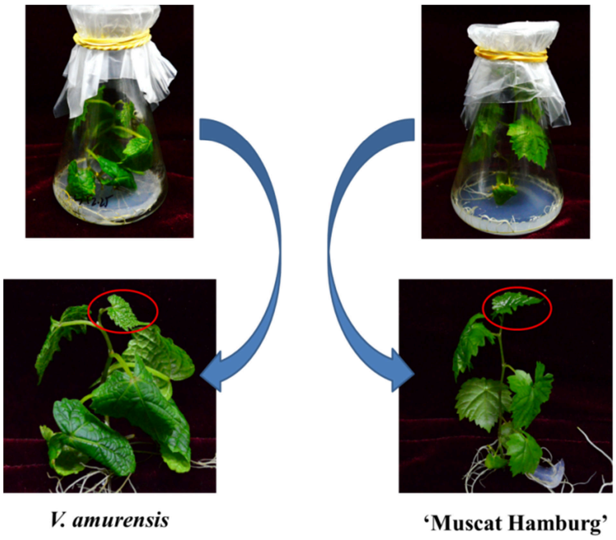

Figure S4 Six-week-old plantlets of *V. amurensis* and 'Muscat Hamburg' used in this study. The shoot apex and one well developed leaf used for RNA extractions were marked with a red ellipse.

Table S1 List of primer sequences used in this study

Primer name	Primer sequence (5'-3')	Description
**Primers used for ORF PCR**		
ERF057-ORF-[illegible]	ATGTGTGGAGGTGCTATC	Forward primer
ERF057-ORF-[illegible]	TTATAGAGCAGCTGAGGTG	Reverse primer
**Primers used for real time RT-PCR**		
ERF057-RT-F	CTATCTCGACGGCAACTCAG	Forward primer
ERF057-RT-R	GAGGAGCAACTGCAAATTCA	Reverse primer
VvActin-F	CTTGCATCCCTCAGCACCTT	Forward primer
VvActin-R	TCCTGTGGACAATGGATGGA	Reverse primer
VvMDH-F	CCATGCATCATCACCCACAA	Forward primer
VvMDH-R	GTCAACCATGCTACTGTCAAAACC	Reverse primer
AtERF13-F	TCCGTTCCTCCCGTTACCT	Forward primer
AtERF13-R	GCCTCCTCCTCACTCCTCTGT	Reverse primer
AtEFR5-F	GGTTGAGAAAGTGTTGAAGACGG	Forward primer
AtEFR5-R	TGGATGAGGAGATAACGGCG	Reverse primer
AtEFR6-F	GTGACGGTGGTTGAGAAAGTGC	Forward primer
AtEFR6-R	CAAACGGTGGGTGTGGAGA	Reverse primer
AtERF105-F	ACTCTAAGCCAACGCAAACCA	Forward primer
AtERF105-R	GCCTAACCAGACACGAACACC	Reverse primer
AtZAT6-F	GAACGGAGGCGGTGTGAG	Forward primer
AtZAT6-R	GGTATCGGCGGTATGTTGAGG	Reverse primer
AtEFR53-F	TAGGAGGGGTGAGTGAGGAGA	Forward primer
AtEFR53-R	TGGTTAGAAATGGGAAAGTGGT	Reverse primer
AtWRKY33-F	GCAGCTCGTGGTAGCGGTT	Forward primer
AtWRKY33-R	TGGTGCTTGAGAAGAAGTAGTGTAGT	Reverse primer
AtWRKY46-F	TTGAGAACGGTGTGTGGAAAA	Forward primer
AtWRKY46-R	ATAAAACCTCGCTGGTAATTGC	Reverse primer
AtACO1-F	AAGAAAATGGGAAATGCGGA	Forward primer
AtACO1-R	GGAAGTGAAGAAAGAAGAGTGAAGTAG	Reverse primer
AtNCED3-F	AAAAGAGAGAGCGAGATTTACCAGT	Forward primer
AtNCED3-R	GCACCCCAAAAGAAACAACAA	Reverse primer
AtbHLH136-F	CTTTCTTCTTGGTCGTTTCCTTTC	Forward primer
AtbHLH136-R	CTCTTTCTTTACTTCTCTCACACTAGCTC	Reverse primer
AtERF104-F	ATGTGGGAGCAGGAGATGGT	Forward primer
AtERF104-R	AGAGTGACGCAAGTGAAAACG	Reverse primer
AtETR2-F	GGTGCAGTTGATTCATGGGA	Forward primer
AtETR2-R	TGTGGGTGAGCGTGGTGG	Reverse primer
AtbHLH113-F	AGGCTGCTTCCTCCTGCTC	Forward primer
AtbHLH113-R	CCATTCCCCTCCTCCTTCTC	Reverse primer
AtWRKY70-F	CATCTCCTCCTCCTCATCCCT	Forward primer
AtWRKY70-R	GGTCCCAATCTTTTCCGACTATC	Reverse primer
AtbZIP61-F	CATCAACGGCAATGTGGGT	Forward primer
AtbZIP61-R	TGTCGTCGTCGCTAAGGCTA	Reverse primer
AtERF43-F	TGGGATTCTCTCTGTTTTTGCT	Forward primer
AtERF43-R	CACTTACATTTCCATCGTCGTTC	Reverse primer
AtbHLH39-F	CGGAGGGAAATGAAATAGACAA	Forward primer
AtbHLH39-R	AGACATGAACGGAGAGATGAGAAC	Reverse primer
AtMYB70-F	TTAAACCGACGGCGAGAGC	Forward primer
AtMYB70-R	GACCCAAGGCAAAGACAACC	Reverse primer
AtPR1-F	TGCAGTGGGACGAGAGGG	Forward primer
AtPR1-R	ATGTTCACGGCGGAGACG	Reverse primer
AtDREB26-F	GTGAGGATGAGAAGTTGGGGA	Forward primer
AtDREB26-R	TTGAGATTGGCTTGAGGGC	Reverse primer
AtPDF1.2b-F	TATCTACGCTGCTCTTGTTCTCTT	Forward primer
AtPDF1.2b-R	GCTGTTTCCGCAAACTCCT	Reverse primer
AtACT2-F	TTACCCGATGGGCAAGTCA	Forward primer
AtACT2-R	AAACGAGGGCTGGAACAAGA	Reverse primer
AtUBQ10-F	CACACTCCACTTGGTCTTGCGT	Forward primer
AtUBQ10-R	TGGTCTTTCCGGTGAGAGTCTTCA	Reverse primer
**Primers used in yeast one-hybrid assay**		
GCC-F	CCCAAGCTTTAAGAGCCGCCTAAGAGCCGCCTAAGAGCCGCCTAAGAGCCGCCCTCGAGCGG	Forward primer
GCC-R	CCGCTCGAGGGCGGCTCTTAGGCGGCTCTTAGGCGGCTCTTAGGCGGCTCTTAAAGCTTGGG	Reverse primer
mGCC-F	CCCAAGCTTTAAGATCCTCCTAAGATCCTCCTAAGATCCTCCTAAGATCCTCCCTCGAGCGG	Forward primer

mGCC-R	CCGCTCGAGGGAGGATCTTAGGAGGATCTTAGGAGGATCTTAGGAGGATCTTAAAGCTTGGG	Reverse primer
DRE-F	CCCAAGCTTTACTACCGACATTACTACCGACATTACTACCGACATTACTACCGACATCTCGAGCGG	Forward primer
DRE-R	CCGCTCGAGATGTCGGTAGTAATGTCGGTAGTAATGTCGGTAGTAATGTCGGTAGTAAAGCTTGGG	Reverse primer
mDRE-F	CCCAAGCTTTACTATTTACATTACTATTTACATTACTATTTACATTACTATTTACATCTCGAGCGG	Forward primer
mDRE-R	CCGCTCGAGATGTAAATAGTAATGTAAATAGTAATGTAAATAGTAATGTAAATAGTAAAGCTTGGG	Reverse primer

Table S2 Significantly differentially expressed genes in *VaERF057* overexpressing Arabidopsis

AGI locus	Symbol	Locus	WT value	VaERF73-OE value	log2(fold_change)	FDR	Significant	Motif number (location) *	
								GCC-box	DRE
**Up-regulated genes**									
AT3G01345	-	3:129137-130749	0.2993	4.9036	4.0342	0.0056	yes		
AT5G39890	PCO2	5:15974392-15976270	0.3976	6.4679	4.0239	0.0056	yes		
AT4G39480	CYP96A9	4:18362257-18364209	0.3189	2.2593	2.8248	0.0244	yes		
AT5G54470	BBX29	5:22114303-22115472	1.2157	8.5292	2.8106	0.0056	yes		
AT1G05575	-	1:1661690-1662187	3.0158	16.5386	2.4552	0.0056	yes		
AT1G04180	YUC9	1:1104622-1105988	0.5023	2.6747	2.4127	0.0498	yes		
AT2G44840	ATERF13	2:18495340-18496272	0.7589	4.0186	2.4047	0.0360	yes		
AT5G47230	ERF5	5:19179938-19181129	3.7241	17.8588	2.2617	0.0056	yes		1 (837)
AT4G25480	DREB1A/CBF3	4:13018213-13019121	2.8959	13.6147	2.2331	0.0056	yes		
AT3G49570	LSU3	3:18372532-18373024	3.0343	13.8132	2.1866	0.0056	yes		
AT2G16060	GLB1	2:6982530-6983631	3.6022	16.3152	2.1793	0.0056	yes		
AT2G15020	-	2:6491433-6493352	1.1767	4.7612	2.0166	0.0056	yes		1 (196)
AT4G17490	ATERF6	4:9752823-9753867	9.4843	37.4082	1.9798	0.0056	yes		1 (452)
AT4G25490	CBF1	4:13021779-13022725	1.0776	4.1205	1.9349	0.0273	yes		
AT3G29000	CML30	3:11005778-11006414	2.9553	11.1131	1.9109	0.0056	yes		1 (781)
AT1G44414	-	1:16847780-16848086	0.0000	2.6314	1.8605	0.0056	yes		
AT5G51190	ERF105	5:20800583-20801416	7.5954	27.4175	1.8519	0.0056	yes		1 (90)
AT4G28720	YUC8	4:14192582-14194256	0.5932	2.0688	1.8023	0.0273	yes		1 (675)
AT1G10560	PUB18	1:3484579-3486965	0.8326	2.8749	1.7878	0.0056	yes		1 (743)
AT5G27420	CNI1	5:9684071-9685561	3.8753	13.3336	1.7827	0.0056	yes		
AT2G24600	-	2:10452115-10454524	2.0584	6.9674	1.7591	0.0102	yes		
AT5G04340	AtZAT6	5:1216130-1217106	6.9904	23.3493	1.7399	0.0056	yes	1 (704)	2 (590, 752)
AT4G16780	ATHB-2	4:9449113-9450743	15.4712	51.4517	1.7336	0.0056	yes		
AT2G20880	ERF053	2:8985972-8987263	1.3663	4.5384	1.7319	0.0056	yes		2 (597, 794)
AT2G38470	WRKY33	2:16108360-16111797	13.0216	42.6359	1.7112	0.0056	yes	1 (746)	
ATCG00920	RRN16S.1	Pt:101011-102502	1.1336	3.7099	1.7105	0.0056	yes		
AT2G46400	WRKY46	2:19043413-19044826	1.0893	3.5118	1.6889	0.0300	yes		1 (507)
AT1G35210	-	1:12896651-12897402	2.0675	6.6205	1.6790	0.0056	yes		1 (847)
AT1G76650	CML38	1:28766749-28768138	8.7692	27.9622	1.6730	0.0056	yes		
AT5G62020	AT-HSFB2A	5:24916088-24917643	1.7653	5.5534	1.6534	0.0056	yes		2 (168, 649)

AT1G72940	-	1:27442237-27443856	5.7711	17.8957	1.6327	0.0056	yes	1 (793)	1 (622)
AT2G40140	CZF1	2:16772072-16774657	10.3269	31.3774	1.6033	0.0056	yes		
AT1G72920	-	1:27437927-27439091	6.9128	20.7881	1.5884	0.0056	yes	1 (788)	1 (618)
AT5G54490	PBP1	5:22121403-22121931	5.8383	17.5502	1.5879	0.0056	yes		1 (674)
AT1G02450	NIMIN1	1:497975-498516	2.7897	8.2494	1.5642	0.0498	yes		1 (294)
AT3G02550	LBD41	3:536504-537998	7.5611	22.3408	1.5630	0.0056	yes		1 (457)
AT1G27730	STZ	1:9648140-9649073	16.3225	48.1982	1.5621	0.0056	yes	1 (669)	1 (717)
AT2G28400	-	2:12148303-12149052	4.3087	12.6876	1.5581	0.0056	yes		
AT2G19590	ACO1	2:8475965-8477502	1.4316	4.1553	1.5374	0.0142	yes		1 (146)
ATCG00950	RRN23S.1	Pt:104690-107500	1.3074	3.5656	1.4475	0.0056	yes	1 (239)	4 (236, 377, 452, 519)
AT1G17147	-	1:5863430-5864009	4.1542	11.1151	1.4199	0.0332	yes		
AT5G41750	-	5:16693908-16698943	1.0726	2.8620	1.4159	0.0056	yes	1 (290)	
AT3G12580	HSP70	3:3991260-3993792	2.2349	5.9529	1.4134	0.0056	yes		1 (949)
AT2G39650	-	2:16527791-16529407	2.4532	6.4801	1.4013	0.0056	yes		1 (49)
AT1G72910	-	1:27435613-27437144	17.0062	43.5665	1.3572	0.0056	yes	1 (810)	
AT3G14440	NCED3	3:4831287-4833599	2.4142	6.1342	1.3453	0.0056	yes		1 (58)
AT1G66090	-	1:24602145-24604763	4.6137	11.4265	1.3084	0.0056	yes		
AT4G02410	LECRK43	4:1059888-1062153	4.2324	10.2991	1.2830	0.0056	yes		
AT5G54610	ANK	5:22184778-22186525	7.6086	18.4732	1.2797	0.0056	yes		
AT3G47420	ATPS3	3:17472341-17475258	2.0491	4.9498	1.2724	0.0056	yes		
AT3G46600	-	3:17157923-17159965	20.6248	49.7930	1.2716	0.0056	yes	1 (886)	
AT1G73870	COL7	1:27779165-27780676	5.5078	13.2585	1.2674	0.0056	yes		
AT1G09350	AtGolS3	1:3019818-3021441	1.6180	3.8571	1.2533	0.0391	yes		4 (181, 209, 605, 616)
AT3G56710	SIB1	3:21006749-21007565	13.5466	32.1341	1.2462	0.0056	yes	1 (721)	3 (135, 182, 498)
ATCG00340	PSAB	Pt:37374-39579	2.5676	6.0654	1.2402	0.0056	yes		
ATCG01210	RRN16S.2	Pt:136146-137637	258.4100	606.9130	1.2318	0.0056	yes		
AT5G57220	CYP81F2	5:23187839-23189857	1.7360	4.0732	1.2304	0.0102	yes		2 (260, 300)
AT5G15120	-	5:4898736-4900581	2.0025	4.6861	1.2266	0.0441	yes		
AT5G39860	PRE1/bHLH136	5:15957416-15958455	11.8565	27.7209	1.2253	0.0056	yes		
AT3G55980	SZF1	3:20776294-20778842	22.1026	51.2023	1.2120	0.0056	yes		
AT1G04770	-	1:1336370-1337849	9.6979	22.1318	1.1904	0.0056	yes		1 (821)
AT5G60900	RLK1	5:24498466-24501494	1.4075	3.2050	1.1872	0.0056	yes		
AT4G25470	CBF2	4:13015276-13016220	2.7421	6.2041	1.1779	0.0418	yes		
AT3G61890	ATHB-12	3:22914153-22915325	2.9438	6.6568	1.1772	0.0214	yes		1 (142)
AT1G16400	CYP79F2	1:5605158-5607473	1.9577	4.3822	1.1625	0.0056	yes	1 (125)	2 (71, 159)
AT4G24570	DIC2	4:12686458-12687650	20.1988	44.9687	1.1547	0.0056	yes		

AT2G26530	AR781	2:11282774-11284229	19.4446	43.0977	1.1482	0.0056	yes	1 (513)	1 (398)
AT5G61600	ERF104	5:24766385-24767430	32.6714	71.8693	1.1374	0.0056	yes		
ATCG01180	RRN23S.2	Pt:131148-133958	258.2490	567.2080	1.1351	0.0056	yes	1 (239)	4 (236, 377, 452, 519)
AT3G23150	ETR2	3:8254662-8257735	2.2312	4.8933	1.1330	0.0056	yes		1 (411)
ATCG00490	RBCL	Pt:54957-56397	17.8428	38.9271	1.1254	0.0056	yes		
AT3G48650	-	3:18024662-18026149	2.2991	4.9340	1.1017	0.0142	yes		
AT1G01260	BHLH13	1:108945-111609	2.1842	4.6663	1.0952	0.0360	yes		
AT2G33380	RD20	2:14144830-14146471	7.1947	15.1857	1.0777	0.0056	yes		2 (458, 565)
AT3G15760	-	3:5337546-5338467	7.0521	14.8535	1.0747	0.0056	yes		1 (735)
AT2G36800	DOGT1	2:15423379-15425016	3.5112	7.3386	1.0635	0.0056	yes		1 (232)
ATCG00020	PSBA	Pt:382-1444	35.0099	72.9502	1.0592	0.0056	yes		1 (694)
AT3G56080	-	3:20810308-20813036	2.8280	5.8835	1.0569	0.0056	yes		
AT5G12050	-	5:3890103-3891513	15.5894	32.0352	1.0391	0.0056	yes		
AT5G20830	SUS1	5:7050225-7055195	7.3059	15.0020	1.0380	0.0056	yes		
AT5G64660	ATCMPG2	5:25841912-25843452	2.7997	5.7416	1.0362	0.0214	yes		
AT1G11050	-	1:3681887-3684169	4.3127	8.8368	1.0349	0.0056	yes	1 (914)	
AT1G74930	ORA47	1:28144131-28145049	4.4509	9.1053	1.0326	0.0391	yes		
AT3G56400	WRKY70	3:20908927-20910481	69.3531	141.7730	1.0316	0.0056	yes		
AT3G27220	-	3:10051611-10053678	3.8009	7.6766	1.0141	0.0056	yes		2 (407, 744)
AT1G13080	CYP71B2	1:4459165-4460940	4.3907	8.8480	1.0109	0.0056	yes		
AT4G32280	IAA29	4:15583386-15584769	6.6877	13.4715	1.0103	0.0056	yes		2 (355, 375)
AT1G32920	-	1:11928806-11929302	71.1725	143.0390	1.0070	0.0056	yes		
ATMG00020	RRN26	Mt:8847-11415	7.9198	15.8437	1.0004	0.0056	yes		
**Down-regulated genes**									
AT5G65080	MAF5	5:25997503-26002465	11.6151	0.8203	-3.8237	0.0056	yes	1 (670)	
AT1G35140	PHI-1	1:12851996-12853128	39.8052	3.3770	-3.5591	0.0056	yes		
AT2G26020	PDF1.2b	2:11089249-11089597	35.7479	3.6227	-3.3027	0.0214	yes	1 (731)	
AT2G39030	AT2G39030/T7F6.20	2:16298226-16299202	4.7820	0.5172	-3.2087	0.0244	yes		2 (76, 270)
AT1G50040	-	1:18542129-18543948	16.4840	2.3485	-2.8112	0.0056	yes		
AT4G16563	-	4:9329656-9331523	22.4411	4.0243	-2.4793	0.0056	yes		1 (198)
AT3G19680	-	3:6840280-6842856	65.7071	12.6862	-2.3728	0.0056	yes		2 (249, 295)
AT4G08950	EXO	4:5740296-5741524	89.2644	19.8846	-2.1664	0.0056	yes		1 (663)
AT5G61160	AACT1	5:24608723-24610304	6.5000	1.5102	-2.1057	0.0056	yes	2 (861, 869)	1 (416)
AT3G49620	DIN11	3:18393746-18396816	6.4623	1.7215	-1.9084	0.0056	yes	1 (497)	
AT2G04033	-	2:1293111-1293488	2.7506	0.0000	-1.9071	0.0214	yes		
AT1G21910	DREB26	1:7696535-7697699	14.8954	4.0686	-1.8723	0.0056	yes		

AT2G14610	PR1	2:6241703-6242463	10.8443	3.2342	-1.7455	0.0056	yes	1 (367)	
AT3G47340	ASN1	3:17437883-17441243	12.1909	3.7471	-1.7020	0.0056	yes		
AT5G25240	-	5:8746604-8747197	28.5624	8.8047	-1.6978	0.0056	yes		
AT5G24030	SLAH3	5:8118316-8122784	13.6103	4.4664	-1.6075	0.0056	yes		
AT2G23290	AtMYB70	2:9904786-9905920	16.0666	5.3209	-1.5943	0.0056	yes		
AT3G56980	BHLH039	3:21086545-21087494	4.6691	1.5478	-1.5930	0.0418	yes	1 (78)	1 (366)
AT5G62920	ARR6	5:25252385-25254212	19.3112	6.4555	-1.5808	0.0056	yes	1 (173)	2 (218, 708)
AT4G14130	XTR7	4:8137050-8138281	6.7449	2.3381	-1.5285	0.0102	yes		
AT2G17230	EXL5	2:7494535-7496016	78.8487	28.1011	-1.4885	0.0056	yes		1 (509)
AT2G23130	AGP17	2:9844298-9845385	79.3155	28.5504	-1.4741	0.0056	yes		
AT5G57550	XTR3	5:23304887-23306421	3.9006	1.4128	-1.4652	0.0471	yes		
AT5G35935	-	5:14083387-14089891	1.4440	0.5265	-1.4556	0.0056	yes		
AT3G45970	ATEXLA1	3:16896165-16898013	88.4778	32.6542	-1.4381	0.0180	yes		1 (740)
AT4G16260	-	4:9200009-9201552	10.6748	3.9842	-1.4218	0.0056	yes	1 (289)	1 (910)
AT1G26260	CIB5	1:9086852-9089378	3.1547	1.1853	-1.4122	0.0441	yes		
AT4G13340	LRX3	4:7758609-7761057	67.8752	26.6012	-1.3514	0.0056	yes		
AT1G76930	ATEXT4	1:28895300-28896883	16.2973	6.4686	-1.3331	0.0056	yes	1 (131)	
AT5G57560	TCH4	5:23307129-23308282	154.0040	61.3058	-1.3289	0.0056	yes		1 (670)
AT4G25810	XTR6	4:13128642-13129884	5.7676	2.2960	-1.3288	0.0244	yes		
AT2G47440	-	2:19469718-19471886	41.2605	16.7755	-1.2984	0.0056	yes		
AT2G19800	MIOX2	2:8530895-8533508	5.4694	2.2504	-1.2812	0.0102	yes		
AT1G76090	SMT3	1:28550408-28551781	65.4704	27.8547	-1.2329	0.0056	yes		
AT2G30930	-	2:13162368-13163398	97.4122	41.6641	-1.2253	0.0056	yes		
AT2G19620	NDL3	2:8483939-8488720	14.4735	6.2891	-1.2025	0.0180	yes		1 (788)
AT4G32800	ERF043	4:15819523-15820871	10.4269	4.6531	-1.1640	0.0056	yes		1 (565)
AT1G11260	STP1	1:3777327-3780337	194.8860	88.7315	-1.1351	0.0056	yes		
AT5G49360	BXL1	5:20011878-20016742	111.3340	50.6932	-1.1350	0.0056	yes	1 (200)	
AT1G10020	-	1:3269603-3271987	27.5670	12.6036	-1.1291	0.0056	yes		1 (384)
AT1G23030	PUB11	1:8156645-8159077	19.0853	8.7952	-1.1177	0.0056	yes		3 (211, 548, 639)
AT1G03300	ATDUF1	1:811032-813086	4.0065	1.8800	-1.0916	0.0273	yes		
AT5G05440	PYL5	5:1609249-1610446	15.2472	7.3022	-1.0622	0.0056	yes		1 (243)
AT4G22513	-	4:11854808-11857313	40.1332	19.2208	-1.0621	0.0056	yes		
AT5G26910	-	5:9465928-9470403	7.6523	3.6924	-1.0514	0.0056	yes		
AT3G54810	BME3-ZF	3:20296231-20298691	35.6879	17.2328	-1.0503	0.0056	yes		
AT5G51550	EXL3	5:20939493-20940895	196.4930	95.2736	-1.0443	0.0056	yes		
AT4G22880	LDOX	4:12004767-12006209	6.5423	3.2083	-1.0280	0.0142	yes		

AT4G28900	-	4:14259140-14263704	2.3376	1.1508	-1.0225	0.0102	yes			
AT3G05727	-	3:1693551-1694138	45.8587	22.7089	-1.0139	0.0056	yes			
AT3G58120	ATBZIP61	3:21520973-21523327	60.3416	29.9590	-1.0102	0.0056	yes			

* Numbers and location of GCC-box or DRE in the 1 kb promoter of each gene.
